# Accurate identification of broadly neutralizing antibodies against dengue virus based on deep stacking strategy with multi-perspective features

**DOI:** 10.1038/s41598-025-31332-3

**Published:** 2025-12-10

**Authors:** Saeed Ahmed, Nalini Schaduangrat, Chonlatip Pipattanaboon, Watshara Shoombuatong

**Affiliations:** 1 Faculty of Medical Technology, Center for Research Innovation and Biomedical Informatics, Mahidol University, Bangkok, 10700 Thailand; 2 Department of Computer Science, University of Swabi, Swabi, 23561 Pakistan; 3 Faculty of Medicine, Department of Microbiology, Khon Kaen University, Khon Kaen, 40002 Thailand

**Keywords:** Dengue virus, Bioinformatics, Ensemble learning, Stacking, Feature selection, Machine learning, Computational biology and bioinformatics, Data mining, Machine learning

## Abstract

Rapid and precise screening for broadly neutralizing antibodies (bNAbs) against all four dengue virus (DENV) serotypes (DENV-1 to DENV-4) is essential to accelerate the development of effective therapeutic antibodies and combat the global burden of dengue fever. However, characterizing bNAbs against DENV through experimental methods remains a significant challenge, as it requires costly laboratory conditions and time-consuming procedures. Therefore, computational methods for in silico identification of bNAbs against DENV based on sequence information are highly desirable and can effectively complement established experimental approaches. Thus, we propose a novel and high-accuracy computational approach, called Deepstack-NAb, for the precise identification of bNAbs against DENV. This method utilizes a stacking ensemble of multiple machine learning (ML) and deep learning (DL) algorithms. First, in Deepstack-NAb, multi-source feature encoding schemes were employed to capture key information from CDR-H3 and epitope data, including conventional feature encodings, natural language processing (NLP)-based feature encodings, and pretrained protein language models (PLMs). Second, all feature descriptors were then fused and optimized using a feature selection technique. Subsequently, well-trained ML and DL methods were constructed to generate multi-perspective features, covering both probabilistic and class information. Finally, the most informative features were selected and employed for optimizing the stacking ensemble model. We conducted comparative experiments to evaluate the performance of Deepstack-NAb against its baseline models and the existing method (i.e., PredNAb). Both cross-validation and independent test results showed that Deepstack-NAb provided a superior performance compared with both its baseline models and PredNAb. On the independent test, Deepstack-NAb achieved an accuracy of 0.905, sensitivity of 0.922, and an MCC of 0.810, representing improvements of 10.34, 13.44, and 20.65%, respectively, over PredNAb. Altogether, the excellent predictive and generalization ability of this new method Deepstack-Nab, highlights its potential as a valuable tool for accurately identifying bNAbs against DENV, significantly reducing false negatives in the process.

**Supplementary Information:**

The online version contains supplementary material available at 10.1038/s41598-025-31332-3.

## Introduction

Dengue virus (DENV) is a significant global health concern, affecting millions of individuals annually across more than 100 countries^1^. It is caused by an RNA virus from the *Flaviviridae* family and is transmitted to humans primarily through the bite of infected *Aedes* mosquitoes, particularily *Aedes aegypti*. DENV comprises of four antigenically distinct serotypes (DENV-1 to DENV-4). While primary infections often result in mild symptoms and induce serotype-specific immunity, secondary infections with a different serotype can lead to severe conditions, such as dengue hemorrhagic fever (DHF) and dengue shock syndrome (DSS)^2^. These severe manifestations are frequently associated with antibody-dependent enhancement (ADE), a phenomenon where pre-existing non-neutralizing antibodies from a prior infection exacerbates the severity of subsequent infections^3^. The global burden of dengue has risen dramatically in recent decades. As of 2024, more than 7.6 million cases and over 3,000 deaths have been reported globally. However, due to underreporting in many endemic regions, the actual burden is likely significantly higher^4^. Despite its widespread impact, there remains no universally effective vaccine or antiviral treatment. The first licensed vaccine, CYD-TDV (Dengvaxia), provides partial protection but has been shown to increase the risk of severe disease in dengue-naive individuals, thereby limiting its use^5^.

Therapeutic antibodies have revolutionized modern medicine, offering precise and effective treatments for a broad spectrum of diseases, including cancer, autoimmune disorders, and infectious diseases^6,7^. These biopharmaceuticals function by specifically binding to antigens on pathogenic organisms or abnormal cells, thereby neutralizing their activity or modulating immune response^7,8^. The structure of an antibody is central to their function, comprising of heavy and light chains organized into Fab and Fc regions^9^. The Fab region, which contains six complementarity determining regions (CDRs), is critical for antigen recognition. Among these, CDR-H3 is the most variable and influential in determining antigen-binding specificity^10,11^. Due to its high variability and functional importance, CDR-H3 has become a central focus in antibody design, engineering, and research^11–13^.

Broadly neutralizing antibodies (bNAbs) that target conserved regions of the DENV envelope protein (E), which has 60–70% conserved sequences across serotypes, have demonstrated significant potential in neutralizing all four DENV serotypes^14,15^. Research efforts have concentrated on targeting specific domains of the E protein, virus’s outer structural component, as prime targets for antibody and vaccine development. These domains include EDI, EDII, EDIII, the EDI-EDII interface, and the EDE region, each of which contains conserved and functionally relevant epitopes^14,16–19^. Among these, EDII and EDIII are particularly important as they contain well-characterized serotype-specific and cross-reactive epitopes, making them ideal targets for bNAbs. For instance, antibodies targeting the fusion loop in EDII have shown broad cross-reactivity, while those directed at EDIII often exhibit strong serotype-specific neutralizing activity^14,18,20,21^. These conserved regions provide a common platform for cross-serotype neutralization and represent critical elements in the design of effective therapeutic strategies and next-generation vaccines.

However, challenges remain due to the risk of ADE, where non-neutralizing or sub-neutralizing antibodies can exacerbate disease severity. To mitigate this, ongoing research focuses on engineering bNAbs that maximize neutralization efficacy while minimizing ADE risks. Recent advancements in antibody discovery methods, such as single B-cell transcriptomics and high-throughput sequencing, are accelerating the identification of potent bNAbs capable of targeting these conserved epitopes with high specificity and therapeutic potential^22,23^. In contrast, traditional antibody discovery methods, such as phage display and yeast surface display, prioritize high binding affinity but are time-consuming and expensive^6^.

Recent advancements in computational tools, including high-throughput sequencing and computational methods, are transforming the discovery process of bNAbs by enabling rapid identification of rare antibody candidates directly from sequence data with high accuracy^24^. Among traditional computational approaches, molecular dynamics (MD) simulations are well-known for antibody identification. However, MD is computationally intensive and may not be suitable in urgent scenarios such as global pandemics. On the other hand, machine learning (ML) and deep learning (DL) approaches offer the capability to identify antibodies using only sequence information, eliminating the need for 3D structural data. This sequence-based design strategy holds substantial promise for large-scale identification of novel bNAbs with therapeutic potential.

Recently, our team proposed the first sequence-based predictor, named PredNAb^25^, for identifying bNAbs against DENV. In PredNAb, both antibody and epitope sequences were encoded using a variety of atom-based, sequence-based, and fingerprint-based feature representation methods, such as Pubchem, EState, MACCS, amino acid composition (AAC), composition-transition-distribution descriptors (CTD), and dipeptide composition (DPC). While this work has greatly advanced the research on the design of CDR-H3-epitope interactions, there are several shortcomings that need to be improved. First, feature representations from natural language processing (NLP)-based feature encodings and pre-trained protein language models (PLMs) have not been previously used in this field. Second, the potential of stacking-based ensemble learning frameworks remains unexplored. Third, the overall prediction performance of PredNAb is not yet satisfactory.

Keeping these limitations in mind, we present Deepstack-NAb, an innovative sequence-based computational approach built upon our previous work to enhance the accurate identification of bNAbs against DENV based on the stacking ensemble method. To the best of our knowledge, this is the first method to apply a stacking ensemble of multiple ML and DL methods for bNAbs identification against DENV. In Deepstack-NAb, we employed multi-source feature encoding schemes to encode CDR-H3 and epitope sequences, covering conventional feature encodings (i.e., blocks substitution matrix (BLOSUM62), amino acid composition (AAC), amphiphilic pseudo-amino acid composition (APAAC), the Composition of Composition, Transition, and Distribution (CTDC), graphical and statistical features (FEAGS), upgraded chaotic game representation (DCGR)), NLP-based feature encoding (i.e., word2vec), and PLM-based feature encoding (i.e., bidirectional encoder representations from transformers (BERT)). Subsequently, we leveraged the strengths of heterogeneous ML and DL methods to construct an ensemble model using the stacking strategy. The major contributions of this study can be summarized as follows. Firstly, we generated multi-perspective features with strong discriminative ability to capture the characteristics and provided key information of bNAbs, including sequential information, graphical information, semantic information, and contextual information. Secondly, the stacking ensemble method leveraged the strengths of heterogeneous ML and DL approaches to deliver a high-accuracy and stable ensemble model. Thirdly, comparative experiments showed that Deepstack-NAb outperformed PredNAb and its baseline models. Impressively, Deepstack-NAb attained a significantly better performance than the compared method in terms of the independent test, with improvements in the area under the receiver operating characteristics (ROC) curve (AUC) of 4.01%, accuracy (ACC) of 10.34%, and Matthew’s correlation coefficient (MCC) of 20.65%, demonstrating its generalization ability and robustness in identifying bNAbs against DENV.

## Materials and methods

### Benchmark dataset

Herein, we utilized the same benchmark dataset introduced in our previous work to optimize the proposed model^25^. In brief, this dataset was constructed from diverse CDR-H3 sequences of antibodies targeting the dengue virus. Each sequence is annotated with its corresponding epitope region on the dengue envelope protein (EDII, EDIII, EDE, interdomain) and its IC50 value. All CDR-H3 sequences adhere to the IMGT numbering standard. Data were derived from in vitro assays (FRNT, PRNT, and ELISA). IC50 values were categorized as neutralizing (≤ 10 µg/ml) or non-neutralizing (> 10 µg/ml), based on a widely accepted potency threshold for neutralizing antibodies. CDR-H3 sequences, epitope regions, and IC50 data were obtained from reputable sources including PubMed and the Google Patent database, selecting only entries with complete CDR-H3 sequences accompanied by epitope mapping and inhibition activity reports. Each CDR-H3 sequence was paired with its corresponding epitope, and IC50 values were categorized using a 10 µg/ml cutoff; values ≤ 10 µg/ml were labelled as neutralizing, while values > 10 µg/ml or antibodies showing no binding were classified as non-neutralizing. All data underwent rigorous preprocessing to handle missing values and remove redundancy. The compilation of CDR-H3–epitope interactions adhered to the principles described by Magar et al.^26^. As a result, the final benchmark dataset includes 1,108 antibody-epitope interactions, consisting of 554 neutralizing and 554 non-neutralizing samples (referred to as 554 positive samples and 554 negative positive samples, respectively). Finally, we randomly selected 80% of all 1,108 samples for the training dataset (443 positive samples and 443 negative positive samples), whereas the remaining 20% (111 positive samples and 111 negative positive samples) were treated as the independent test dataset. In the meanwhile, we applied Euclidean distance in conjunction with dipeptide composition (DPC) to compute the similarity measurement among sequence pairs in the training and independent test datasets. To elucidate sequence redundancy, we examined the cumulative fractions of sequence pairs exceeding multiple similarity cut-offs (i.e., 0.5, 0.6, 0.7, 0.8, and 0.9). As shown in Supplementary Figure S1 and Table S1, it can be observed that 99.99%, 99.89%, 98.63%, 76.13%, and 54.13% of sequence pairs displayed similarity scores of < 0.9, < 0.8, < 0.7, < 0.6, and < 0.5, respectively, indicating substantial diversity between the training and independent test datasets.

### Feature extraction scheme

Numerous studies have demonstrated that combining multiple types of feature descriptors can attain improved performance compared to using single-based feature descriptors^27–33^. Therefore, we employed eight feature encodings, including conventional feature encodings (i.e., BLOSUM62, AAC, APAAC, CTDC, FEAGS, and DCGR), NLP-based feature encoding (i.e., word2vec), and PLM-based feature encoding (i.e., BERT), to convert the CDR-H3 and epitope sequences into numerical feature vectors. The choice of these feature encodings was made based on the objective of integrating multi-source feature encodings with diverse characteristics and perspectives. For conventional feature encodings, AAC, APAAC, and CTDC are deemed as classical feature encoding methods that describes the compositions of 20 amino acids, the combined information of the compositions of 20 amino acids and the sequence-order, and the percentage frequency of multiples amino acid property groups, respectively^28,31–34^. FEAGS, developed by Mu et al.^35^, leverages both physicochemical properties of amino acids and statistical features of protein sequences. In FEAGS, AAindex was employed to numerically extract physiochemical properties (PP) with 158 indices selected out of 566 available indices to construct the final feature vector by combining them with statistical features. BLOSUM62 provides evolutionary information in the form of a position-specific scoring matrix (PSSM)^36^, enabling the calculation of sequence similarity and identification of conserved regions across protein families^27,30^. Furthermore, inspired by the classical chaos game representation (CGR) technique widely applied in DNA sequence characterization^37,38^, Mu et al. proposed an updated method termed DCGR, which incorporates physicochemical properties and distributional information^39^. The dimensions of the feature vectors based on BLOSUM62, AAC, APAAC, CTDC, FEAGS, and DCGR are 20 $\times$ 2, 20 $\times$ 2, 22 $\times$ 2, 39 $\times$ 2, 578 $\times$ 2, and 50 $\times$ 2, respectively. In recent years, NLP-based word embedding methods have been successfully applied to create high-dimensional embeddings of biological sequences^40^. In the bioinformatics field, word embedding methods (such as word2vec and fastText) have proven effective in capturing semantic information and preserving local amino acid residue patterns better than traditional sequence-based feature encodings^41–43^. Therefore, we applied the word2vec embedding method to encode the entire CDR-H3 and epitope sequences in this study. Another important advancement in applying NLP to bioinformatics is the development of protein language models (PLMs). The PLMs has been successful in providing representations (embeddings) that capture crucial information, including contextual information, protein semantic information, and residue-wise level details^44,45^. Among various PLMs, we applied the Bidirectional Encoder Representations from Transformers (BERT) model^46^ released by Zhang et al.^47^ to represent the CDR-H3 and epitope sequences into numerical feature vectors. The dimensions of the feature vectors based on word2vec and BERT are 220 $\times$ 2 and 768 $\times$ 2, respectively.

### Feature selection method

Least absolute shrinkage and selection operator algorithm (Lasso) is a well-regarded embedded feature selection method, originally proposed by Robert Tibshirani in 1996^48^. In this algorithm, a set of important features are selected by applying the use of a simple method (i.e., least squares regression)^49^. However, a drawback of the original lasso is that it cannot capture information regarding connected features. In 2006, the group lasso was introduced to address this issues by integrating important constraints that enable feature selection at the group level^50^. This approach utilizes additional constraints by leveraging sets of coefficient vectors, thereby enhancing the sparsity of feature groups. In particular, each single feature is deemed as a set of coefficients. As a result, if the sets’ coefficients are non-zero, the corresponding features are selected and included in the optimal feature subset; otherwise, the corresponding features are discarded. In general, the group lasso is formulated as follows:

1 $${\mathrm{arg}\mathit{min}}_{\beta }\frac{1}{2}\sum_{i=1}^{n}{\Vert Y-\sum_{l=1}^{L}{X}_{l}{\beta }_{l}\Vert }_{2}^{2}+\propto \sum_{l=1}^{L}\Vert {\beta }_{l}\Vert$$

Here, $X$ is the matrix of $n$ samples and $m$ features and ${X}_{l}$ is the submatrix of $n$ samples and $l$ feature groups, while $Y$ is the *n*D vector of observations and $\beta$ is represented as an *m*D coefficient vector. In the group lasso, the *m* features are divided into $L$ groups, while ${X}_{l}$ represents the sign matrix matching the $l$ group having ${\beta }_{l}$ coefficient vector. Herein, the regularization parameter α was empirically set to 0.03 based on preliminary testing. After applying the approach, the number of selected features with non-zero weight are 279. Until now, numerous studies have elucidated that group lasso is capable of enhancing the predictive performance in several biological and chemical classification problems^51–53^.

### Deep stacking ensemble learning framework

**Fig. 1:**
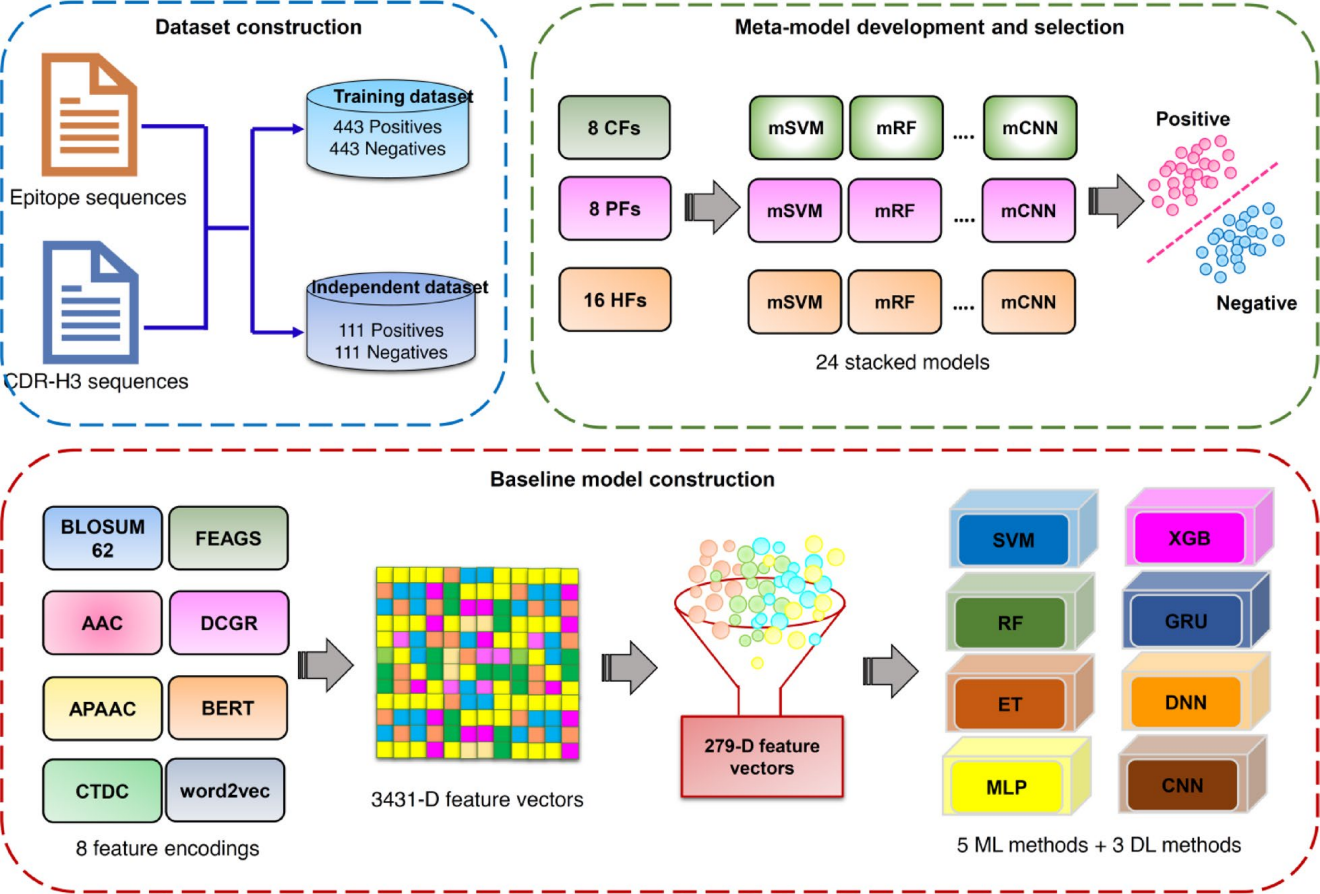
Workflow of Deepstack-NAb development. (i) Collection of the benchmark dataset for training and testing the proposed model, where positive and negative samples are neutralizing and non-neutralizing samples, respectively. (ii) Construction of baseline models using different ML and DL methods in conjunction with the optimal feature subset. (iii) Development of various meta-models using different ML and DL methods in conjunction with three new feature representations.

We employed the stacking ensemble learning technique to develop Deepstack-NAb in order to accurately identify bNAbs against DENV. This technique contains two main phases and the corresponding models developed at each phase are referred to as baseline models and meta-model, respectively^54^. In the stacking strategy, a pool of baseline models is first used to make predictions on the input data. These prediction outcomes from the baseline models are treated as features to develop the stacked model. The advantage of this strategy is the ability to automatically leverage the strengths of different prediction methods, thereby improving overall predictive capability^29,30,32,33,55–57^. The overall framework of the proposed model, Deepstack-NAb is summarized in Fig. 1, while the development of both baseline and meta-models is described below.

In this study, multiple ML and DL methods (i.e., extremely randomized trees (ERT), multilayer perceptron (MLP), random forest (RF), extreme gradient boosting (XGB), support vector machine (SVM), deep neural networks (DNN), gated recurrent unit (GRU), and convolutional neural network (CNN)) were selected and used to develop the baseline models. The reason for selecting heterogeneous ML and DL methods was to obtain an ensemble model with improved accuracy and generalization capability^57,58^. Rather than using a single type of feature descriptor, we combined eight types of feature encodings to create a fused feature vector (Fusion) that captured comprehensive information from CDR-H3 and epitope sequences to construct the baseline models. After that, an optimal feature subset was determined using the group lasso to select *m* important features, which were used to develop eight well-trained baseline models. Finally, 8 baseline models were developed herein. In the second phase, the outcomes predicted by all the baseline models were applied as features and served as input for training the meta-model. These outcomes provide two types of feature representations, covering class (CF) and probabilistic (PF) features. For a given sample *P*, both PFs and CFs were represented as 8-D feature vectors. To maximize the utility of these new feature representations, we combined 8 PFs and 8 CFs to generate a hybrid feature vector (HF), which was represented by a 16-D feature vector. Then, several meta-models were trained using CF, PF, and HF in conjunction with the same collection of ML and DL methods used in the first phase. Herein, the hyperparameters for both baseline models and meta-models were optimized based on the grid search method coupled with the cross-validation scheme, while the Scikit-learn library v0.24.1 package^59^ was used to implement all baseline models and meta-models developed herein. The grid search space for each method is recorded in Supplementary Table S2. The stacked model having the highest cross-validation MCC was deemed as the best-performing model and applied to optimize the proposed model.

### Model evaluation

The performance evaluation of the proposed models is a crucial step to assess their effectiveness and robustness in precisely identifying new samples. Herein, we utilized two standard validation strategies, including ten-fold cross-validation and independent tests. It is important to note that the samples in the independent test dataset were not found in the training dataset to ensure an unbiased assessment of the model’s generalization ability. All baseline models and stacked models were evaluated for their performance by conducting the ten-fold cross-validation and independent tests on the training and independent datasets, respectively. In general, the ten-fold cross-validation procedure involves ten individual training sessions. For each session, nine-tenth of the dataset is selected as the sub-training set, and the remaining data (or the sub-testing set) is used for the performance evaluation. As a result, ten different prediction results are obtained and averaged to determine the prediction result. To comprehensively measure model performance, several well-regarded performance measures were used, including AUC, MCC, F1, sensitivity (SN), and specificity (SP)^29,32,60–66^, and these performance measures are defined as follows:

2 $$\mathrm{MCC}=\frac{\mathrm{TP}\times \mathrm{TN}-\mathrm{FP}\times \mathrm{FN}}{\sqrt[]{(\mathrm{TP}+\mathrm{FP})(\mathrm{TP}+\mathrm{FN})(\mathrm{TN}+\mathrm{FP})(\mathrm{TN}+\mathrm{FN})}}$$

3 $$\mathrm{F}1=2\times \frac{\mathrm{TP}}{2\mathrm{TP}+\mathrm{FP}+\mathrm{FN}}$$

4 $$\mathrm{ACC}=\frac{\mathrm{TP}+\mathrm{TN}}{\left(\mathrm{TP}+\mathrm{TN}+\mathrm{FP}+\mathrm{FN}\right)}$$

5 $$\mathrm{SN}=\frac{\mathrm{TP}}{\left(\mathrm{TP}+\mathrm{FN}\right)}$$

6 $$\mathrm{SP}=\frac{\mathrm{TN}}{\left(\mathrm{TN}+\mathrm{FP}\right)}$$

where $\mathrm{TP}$ and $\mathrm{TF}$ signify the numbers of true positives and true negatives, respectively. And, $\mathrm{FN}$ and $\mathrm{FP}$ signify the numbers of false negatives and false positives, respectively^67–69^.

## Results and discussion

### Performance evaluation of different baseline models

**Fig. 2:**
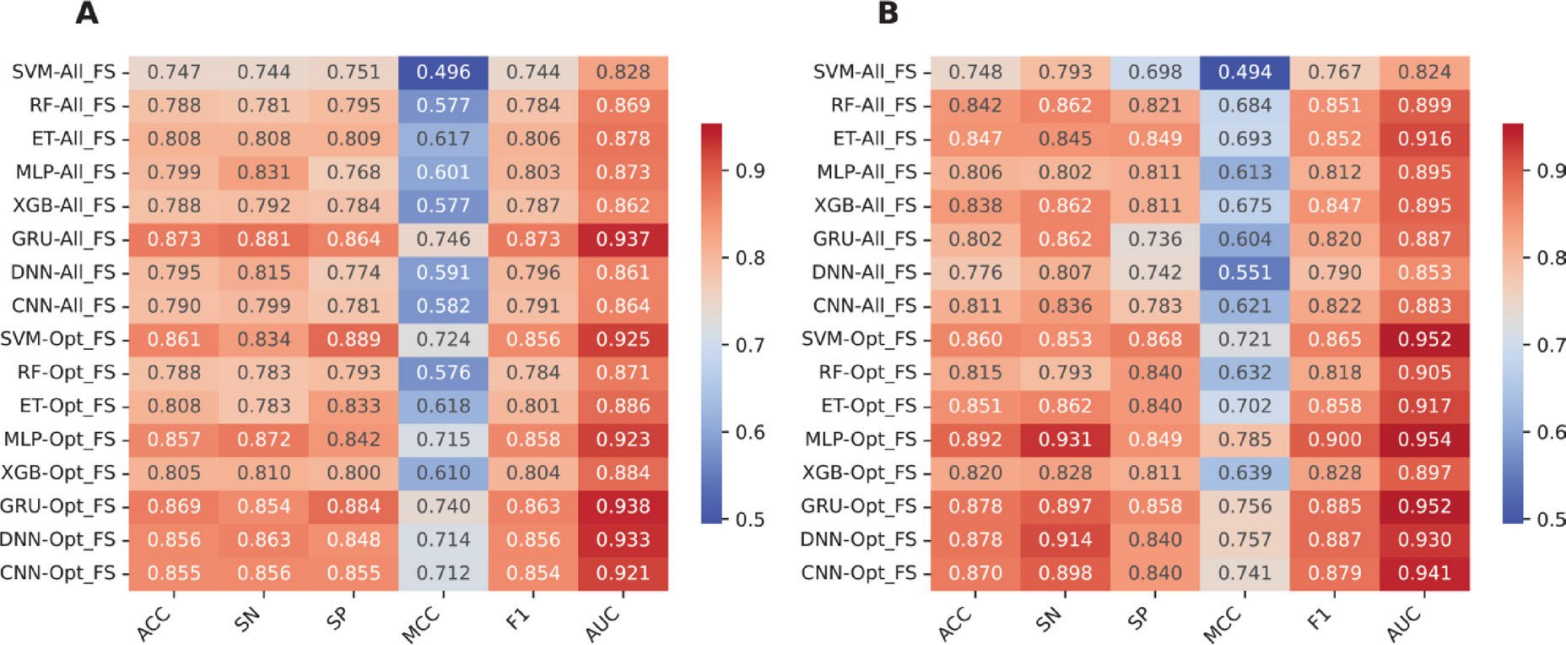
Performance comparison of baseline models trained with all features (All_FS) and optimal feature subset (Opt_FS) over the cross-validation (**A**) and independent tests (**B**).

This section presents the comparative experiment results of various baseline models (i.e., SVM, RF, ET, MLP, XGB, GRU, DNN, and CNN) for identifying bNAbs against DENV. All baseline models were built using the Fusion feature set and assessed based on their prediction performance over both the ten-fold cross-validation and independent tests. Figure 2 and Table 1 summarize the performance evaluation results for all eight baseline models. From the results listed in Table 1, it can be perceived that five out of eight baseline models (i.e., GRU, ET, MLP, DNN, and CNN) showed relatively strong predictive performance. In addition, among these five baseline models, the SVM baseline model exhibited MCC of less than 0.5 in terms of both the cross-validation and independent tests. A possible reason for the obtained result is the high dimensionality of the Fusion feature space (i.e., a 3431D feature vector), which exceeds the number of the training samples (i.e., 886 samples). This imbalance between feature dimension and sample size, known as the feature dimensionality issue, can degrade the model performance by causing overfitting and reducing generalization capability.

**Table 1:** Performance evaluation of baseline models trained with All_FS and Opt_FS over the cross-validation test.

Method	Feature set	ACC	SN	SP	MCC	F1	AUC
ET	All_FS	0.808	0.808	0.809	0.617	0.806	0.878
	Opt_FS	0.808	0.783	0.833	0.618	0.801	0.886
MLP	All_FS	0.799	0.831	0.768	0.601	0.803	0.873
	Opt_FS	0.857	0.872	0.842	0.715	0.858	0.923
RF	All_FS	0.788	0.781	0.795	0.577	0.784	0.869
	Opt_FS	0.788	0.783	0.793	0.576	0.784	0.871
SVM	All_FS	0.747	0.744	0.751	0.496	0.744	0.828
	Opt_FS	0.861	0.834	0.889	0.724	0.856	0.925
XGB	All_FS	0.788	0.792	0.784	0.577	0.787	0.862
	Opt_FS	0.805	0.810	0.800	0.610	0.804	0.884
CNN	All_FS	0.790	0.799	0.781	0.582	0.791	0.864
	Opt_FS	0.855	0.856	0.855	0.712	0.854	0.921
DNN	All_FS	0.795	0.815	0.774	0.591	0.796	0.861
	Opt_FS	0.856	0.863	0.848	0.714	0.856	0.933
GRU	All_FS	0.873	0.881	0.864	0.746	0.873	0.937
	Opt_FS	0.869	0.854	0.884	0.740	0.863	0.938

To solve this issue, we employed the LASSO algorithm for selecting *m* out of 3431 features to enhance the performance of the baseline models. After selecting important features from the fused features using LASSO, the original feature vector was reduced to 279D from 3431D, which were referred to as Opt_FS and All_FS herein, respectively. To evaluate the impact of feature selection, we compared the performance of all baseline models trained with All_FS and Opt_FS, in terms of MCC. Among the eight baseline models, the MCC for SVM, CNN, DNN, MLP, and XGB baseline models trained with Opt_FS achieved improved results compared to All_FS over the cross-validation test, providing improvements of 22.84 (0.496 versus 0.724), 13.00 (0.582 versus 0.724), 12.28 (0.591 versus 0.714), 11.37 (0.601 versus 0.715), and 3.26 (0.577 versus 0.610)%, respectively (Table 1). Furthermore, the ACC, SN, SP, F1, and AUC of these five baseline models trained with Opt_FS were 1.69–11.40, 1.76–8.96, 1.55–13.81, 3.26–22.48, 1.65–11.19 and 2.21–9.63%, respectively, higher than that of All_FS. For the remaining baseline models, although their performance based on Opt_FS was comparable or slightly reduced in terms of MCC compared with the models trained with All_FS (i.e., 0.617 versus 0.618 for RF and 0.746 versus 0.740 for GRU), using Opt_FS can reduce computational cost. These results demonstrated that Opt_FS is capable of improving the prediction performance. Thus, Opt_FS was employed for the construction of our final model.

### Optimization of Deepstack-NAb using class and probabilistic information

**Fig. 3:**
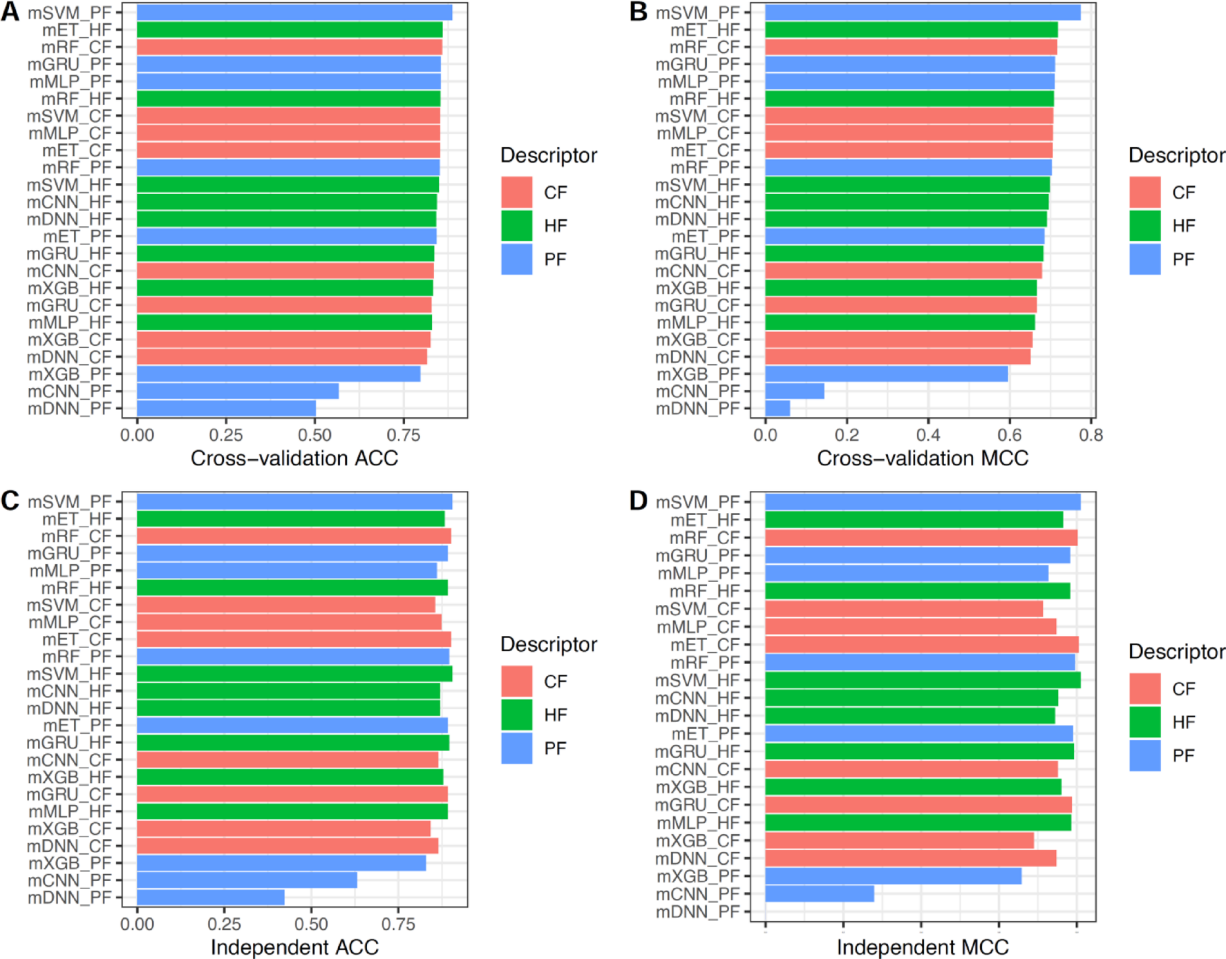
ACC and MCC of different stacked models trained with three types of feature representations over the cross-validation (**A-B**) and independent tests (**C-D**).

Among all eight baseline models, the model trained using GRU outperformed the others, achieving MCC scores of 0.740 and 0.756 over the the ten-fold cross-validation and independent tests, respectively. However, its overall performance remains unsatisfactory. To achieve a higher prediction performance, various ML and DL algorithms (i.e., mET, mMLP, mRF, mXGB, mSVM, mCNN, mDNN, and mGRU) were applied as meta-models to train the stacked models, where corresponding meta-models are ET-based, MLP-based, RF-based, XGB-based, SVM-based, CNN-based, DNN-based, and GRU-based meta-models, respectively. In this section, we display the experimental evaluation of several stacked models in identifying bNAbs against DENV. Each stacked model was trained with CF, PF, and HF. The CF, PF, and HF were represented with 8D, 8D, and 16D feature vectors, respectively. In total, 24 stacked models were evaluated, with their performance metrics provided in Fig. 3 and Table 2. Overall, we observed that mSVM_PF, mET_HF, mRF_CF, mGRU_PF, mMLP_PF were the top-five stacked models, which provided the highest cross-validation MCC of 0.774, 0.718, 0.716, 0.711, 0.710, respectively. Among the top-five stacked models, it is clear that mSVM_PF outperformed other compared models in terms of ACC, MCC, and F1 over both the the ten-fold cross-validation and independent tests. To be specific, the ACC, MCC, and F1 (cross-validation, independent test) of mSVM_PF were (0.887, 0.905), (0.774, 0.810), and (0.894, 0.911), respectively. This implies that the stacking-based ensemble learning framework could enhance predictor performance. Accordingly, mSVM trained using PF was selected for the construction of our final model (Deepstack-NAb).

**Table 2:** Performance evaluation of stacked models trained with various multi-perspective features over the cross-validation and independent tests.

Feature	Classifier	Cross-validation test				Independent test			
		ACC	MCC	F1	AUC	ACC	MCC	F1	AUC
PF	mET	0.842	0.685	0.840	0.919	0.892	0.790	0.903	0.956
	mMLP	0.854	0.710	0.851	0.896	0.860	0.727	0.876	0.938
	mRF	0.851	0.703	0.846	0.917	0.896	0.795	0.905	0.951
	mXGB	0.797	0.595	0.794	0.885	0.829	0.658	0.833	0.930
	mSVM	0.887	0.774	0.894	0.911	0.905	0.810	0.911	0.925
	mCNN	0.567	0.144	0.436	0.582	0.631	0.279	0.594	0.757
	mDNN	0.503	0.060	0.448	0.619	0.423	-0.212	0.045	0.144
	mGRU	0.854	0.711	0.856	0.873	0.892	0.783	0.897	0.941
CF	mET	0.852	0.705	0.851	0.899	0.901	0.805	0.910	0.946
	mMLP	0.852	0.706	0.854	0.895	0.874	0.748	0.883	0.880
	mRF	0.858	0.716	0.857	0.879	0.901	0.802	0.907	0.933
	mXGB	0.825	0.655	0.812	0.865	0.842	0.690	0.840	0.916
	mSVM	0.852	0.707	0.852	0.890	0.856	0.714	0.869	0.880
	mCNN	0.834	0.679	0.845	0.917	0.865	0.751	0.885	0.948
	mDNN	0.815	0.651	0.834	0.901	0.865	0.748	0.884	0.934
	mGRU	0.828	0.666	0.839	0.899	0.892	0.788	0.902	0.950
HF	mET	0.859	0.718	0.855	0.916	0.883	0.765	0.888	0.945
	mMLP	0.830	0.662	0.833	0.897	0.892	0.785	0.900	0.950
	mRF	0.853	0.708	0.853	0.913	0.892	0.783	0.897	0.959
	mXGB	0.832	0.666	0.829	0.899	0.878	0.760	0.879	0.948
	mSVM	0.849	0.698	0.848	0.886	0.905	0.810	0.911	0.958
	mCNN	0.843	0.695	0.849	0.912	0.869	0.752	0.886	0.948
	mDNN	0.841	0.691	0.851	0.900	0.869	0.744	0.883	0.947
	mGRU	0.836	0.683	0.846	0.915	0.896	0.793	0.903	0.964

### Comparison of Deepstack-NAb with its constituent base-classifiers

**Fig. 4:**
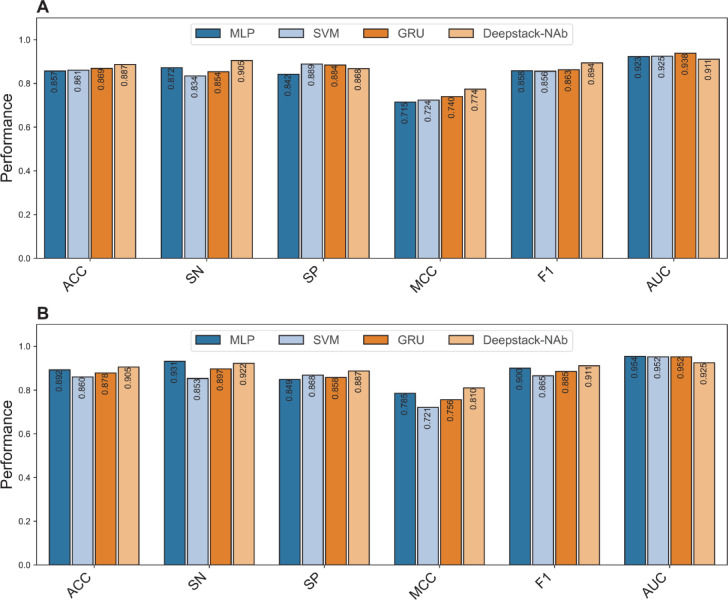
Performance comparison of Deepstack-NAb with top-three baseline models over the cross-validation (**A**) and independent tests (**B**).

It is evident from Table 1 that, in terms of baseline models over the cross-validation test, GRU achieved the highest MCC of 0.740, while SVM and MLP provide the second- and third-highest MCC of 0.724 and 0.715, respectively. Thus, to illustrate whether the stacking ensemble method plays an important role in providing performance improvement, we compared our developed stacking ensemble model Deepstack-Nab, against these top-three baseline models. As shown in Fig. 4 and Table 3, we observe that Deepstack-NAb outperforms the top-three baseline models in terms of ACC (0.887 versus 0.857–0.869), SN (0.905 versus 0.834–0.872), MCC (0.774 versus 0.715–0.740), and F1 (0.894 versus 0.856–0.858) over the cross-validation test. This means that Deepstack-NAb achieved improvements of 1.81–0.307, 3.32–7.16, 3.41–5.98, and 3.06–3.78% in ACC, SN, MCC, and F1, respectively. For the independent test, Deepstack-NAb still provided superior predictive performance compared to the top-three baseline models, with improvements of 1.35–4.50, 1.89–3.77, 2.58–8.97, and 1.06–4.60% in ACC, SP, MCC, and F1, respectively. Impressively, when compared with all the eight baseline models, the MCC of Deepstack-NAb was 3.41–19.82 and 2.58–17.83% as judged by the cross-validation and independent tests, respectively. Altogether, Deepstack-NAb can effectively achieve more accurate and stable identification of bNAbs against DENV, confirming the role of the stacking ensemble method in enhancing prediction performance.

**Table 3:** Performance comparison of Deepstack-NAb with its baseline models over the cross-validation and independent tests.

Evaluation strategy	Method	ACC	SN	SP	MCC	F1	AUC
Cross-validation	RF	0.788	0.783	0.793	0.576	0.784	0.871
	XGB	0.805	0.810	0.800	0.610	0.804	0.884
	ET	0.808	0.783	0.833	0.618	0.801	0.886
	CNN	0.855	0.856	0.855	0.712	0.854	0.921
	DNN	0.856	0.863	0.848	0.714	0.856	0.933
	MLP	0.857	0.872	0.842	0.715	0.858	0.923
	SVM	0.861	0.834	0.889	0.724	0.856	0.925
	GRU	0.869	0.854	0.884	0.740	0.863	0.938
	Deepstack-NAb	0.887	0.905	0.868	0.774	0.894	0.911
Independent test	RF	0.815	0.793	0.840	0.632	0.818	0.905
	XGB	0.820	0.828	0.811	0.639	0.828	0.897
	ET	0.851	0.862	0.840	0.702	0.858	0.917
	CNN	0.870	0.898	0.840	0.741	0.879	0.941
	DNN	0.878	0.914	0.840	0.757	0.887	0.930
	MLP	0.892	0.931	0.849	0.785	0.900	0.954
	SVM	0.860	0.853	0.868	0.721	0.865	0.952
	GRU	0.878	0.897	0.858	0.756	0.885	0.952
	Deepstack-NAb	0.905	0.922	0.887	0.810	0.911	0.925

### Analysis of our multi-perspective features

**Fig. 5:**
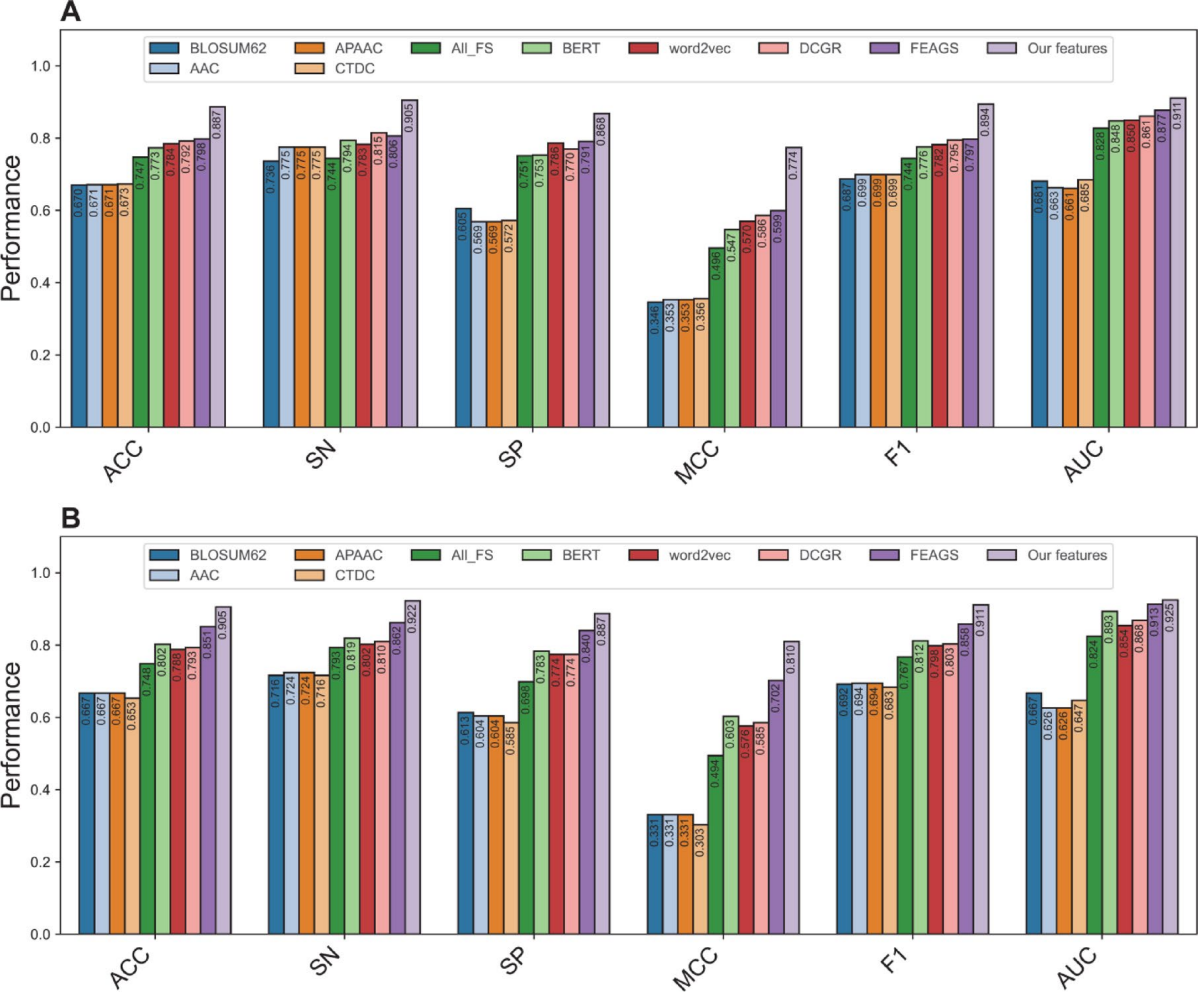
Performance comparison of our multi-perspective features with conventional feature descriptors (i.e., BLOSUM62, AAC, APAAC, CTDC, FEAGS, DCGR, word2vec, and BERT) over the cross-validation (**A**) and independent tests (**B**).

**Fig. 6:**
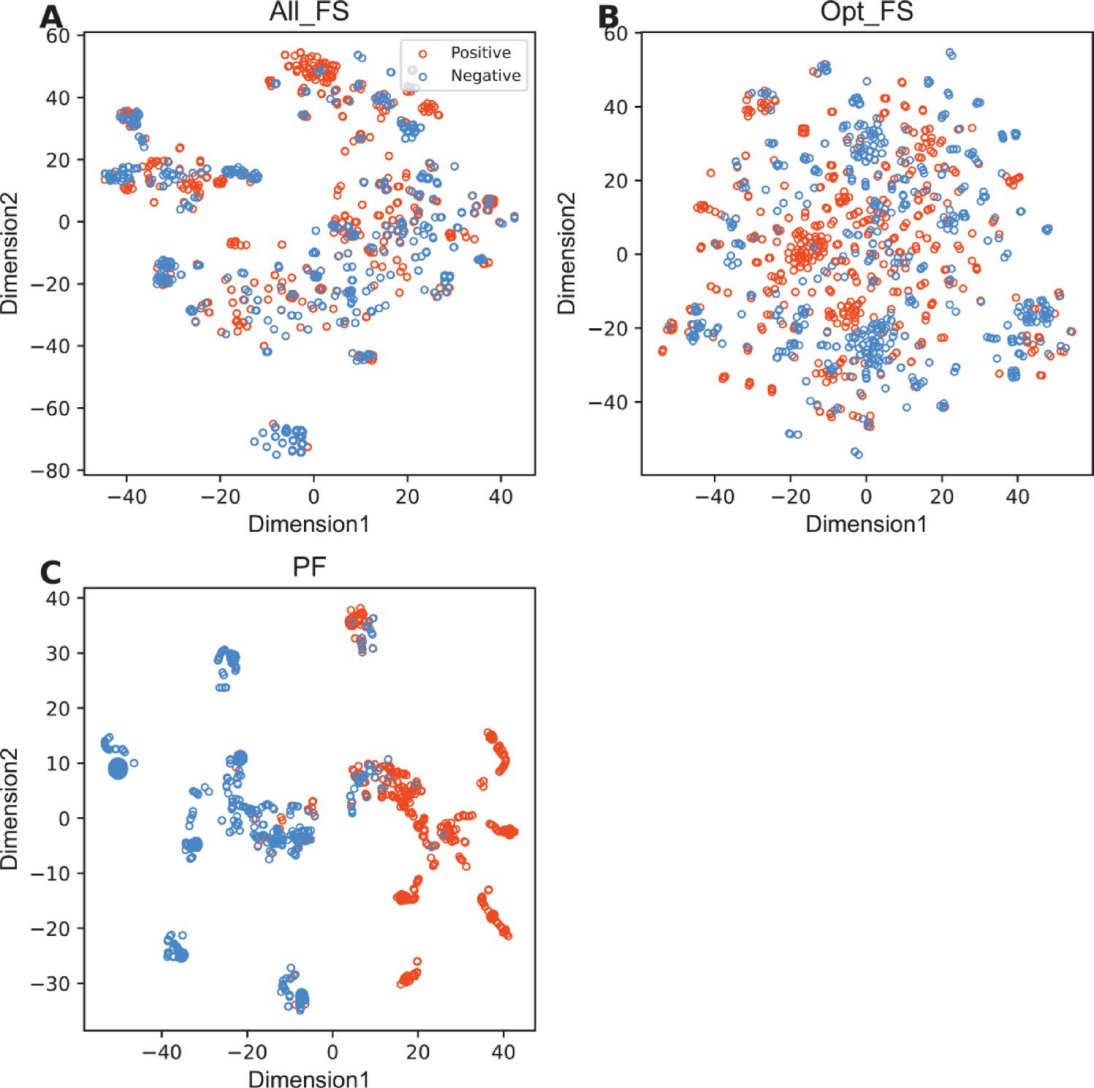
t-SNE visualizations of different feature representations (i.e., All_FS, Opt_FS, and PF). In two-dimensional space, where positive and negative samples are neutralizing and non-neutralizing samples, respectively.

In this section, we investigated whether our proposed multi-perspective features (i.e., PF) play a pivotal role in enhancing both the feature representation capability and the prediction performance of bNAbs against DENV. To demonstrate this, we initially compared the prediction results obtained using PF to those obtained using conventional feature descriptors (i.e., BLOSUM62, AAC, APAAC, CTDC, FEAGS, DCGR, word2vec, and BERT). For a fair comparison, PF and all the conventional feature descriptors were individually input into the SVM model, and their prediction results were assessed based on the ten-fold cross-validation and independent tests. Figure 5 and Table 4 show the prediction results obtained using PF and the compared features. We can observe that PF exhibited higher feature ability across all the six performance measures over both standard validation strategies. Specifically, in terms of the independent test, the MCC, ACC, and F1 scores of PF were approximately 10.85–47.98, 5.41–23.87, and 5.23–21.90% higher, respectively, than those of the compared features. To further confirm the feature representation ability of PF, we applied the popular dimensionality reduction approach (named t-SNE^70,71^), to visualize and compare the discriminative ability of PF against All_FS and Opt_FS along with the conventional feature descriptors. Herein, each feature set was projected into a 2D vector scatter plot based on t-SNE approach. Then, all nine 2D vector scatter plots from PF and the conventional feature descriptors are shown in Fig. 6 and Supplementary Figure S2. The scatter plot generated by PF was easily distinguishable and provided clear clusters among the two classes. Altogether, it can be inferred that our proposed PF features had great discriminative ability in capturing information related to bNAbs against DENV, leading to potentially improved predictive performance.

**Table 4:** Performance comparison of our multi-perspective features with conventional feature descriptors over the cross-validation and independent tests.

Evaluation strategy	Feature	ACC	SN	SP	MCC	F1	AUC
Cross-validation	AAC	0.671	0.775	0.569	0.353	0.699	0.663
	APAAC	0.671	0.775	0.569	0.353	0.699	0.661
	CTDC	0.673	0.775	0.572	0.356	0.699	0.685
	FEAGS	0.798	0.806	0.791	0.599	0.797	0.877
	DCGR	0.792	0.815	0.770	0.586	0.795	0.861
	BLOSUM62	0.670	0.736	0.605	0.346	0.687	0.681
	word2vec	0.784	0.783	0.786	0.570	0.782	0.850
	BERT	0.773	0.794	0.753	0.547	0.776	0.848
	Our multi-perspective features	0.887	0.905	0.868	0.774	0.894	0.911
Independent test	AAC	0.667	0.724	0.604	0.331	0.694	0.626
	APAAC	0.667	0.724	0.604	0.331	0.694	0.626
	CTDC	0.653	0.716	0.585	0.303	0.683	0.647
	FEAGS	0.851	0.862	0.840	0.702	0.858	0.913
	DCGR	0.793	0.810	0.774	0.585	0.803	0.868
	BLOSUM62	0.667	0.716	0.613	0.331	0.692	0.667
	word2vec	0.788	0.802	0.774	0.576	0.798	0.854
	BERT	0.802	0.819	0.783	0.603	0.812	0.893
	Our multi-perspective features	0.905	0.922	0.887	0.810	0.911	0.925

### Comparison of Deepstack-NAb with the existing method

In this section, we utilized the same dataset previously used by PredNAb^25^ to assess and compare the performance of Deepstack-NAb against PredNAb. The performance comparison results are recorded in Table 5, and it is important to note that the predictive results for the compared method were directly obtained from Natsrita et al.^25^. As can be seen from Table 5, Deepstack-NAb shows superior predictive performance as indicated by all the five performance measures (i.e., ACC, AUC, MCC, SP, and SN) over both standard validation strategies. Remarkably, in terms of the independent test, Deepstack-NAb showed substantial improvements of 10.34% (0.802 versus 0.905) in ACC along with 13.44% (0.788 versus 0.922) in SN, and 20.65% (0.604 versus 0.810) in MCC. Overall, it can be concluded that our proposed method, termed Deepstack-NAb, displayed a robust and stable predictive performance, highlighting its effectiveness and generalization ability. Furthermore, the excellent SN and MCC of Deepstack-NAb over the independent test is sufficient to indicate that this method can be effectively applied for accurate in silico identification of bNAbs against DENV.

**Table 5:** Performance comparison of Deepstack-NAb with the existing method over the cross-validation and independent tests.

Evaluation strategy	Method	ACC	SN	SP	MCC	AUC
Cross-validation	PredNAb	0.815	0.791	0.842	0.630	0.879
	Deepstack-NAb	0.887	0.905	0.868	0.774	0.911
Independent test	PredNAb	0.802	0.788	0.817	0.604	0.885
	Deepstack-NAb	0.905	0.922	0.887	0.810	0.925

## Conclusion

This study presents a novel stacking ensemble learning method, named Deepstack-NAb, aimed at achieving more precise and improved identification of bNAbs against DENV. Deepstack-NAb combined five different ML models (i.e., SVM, RF, ET, MLP, and XGB) and three different DL models (i.e., GRU, DNN, and CNN) to form a high-accuracy and stable ensemble model using the stacking strategy. In addition, we proposed multi-perspective features that were capable of capturing crucial information related to bNAbs, including sequential information, graphical information, semantic information, and contextual information. Through a series of comparative experiments, Deepstack-NAb consistently outperformed its baseline models and PredNAb, achieving excellent performance measures in terms of ACC, SN, MCC, and F1 over both the cross-validation and independent tests. Specifically, Deepstack-NAb achieved an ACC of 0.905, SN of 0.922, and MCC of 0.810 over the independent test, providing improvements of 10.34, 13.4, and 20.65%, respectively, compared to PredNAb. Furthermore, we compared our multi-perspective features with conventional feature descriptors, and the comparative results demonstrated that our multi-perspective features had outstanding discriminative power in capturing the characteristic of bNAbs against DENV. Deepstack-NAb provided outstanding performance compared to PredNAb for the following reasons: (i) We utilized different feature encoding methods from multi sources to capture crucial information from CDR-H3 and epitope sequences, and (ii) Unlike the single model-based approach used in PredNAb, we developed a stacked ensemble learning model that leverages the strengths of multiple ML and DL methods. While Deepstack-NAb shows great potential in reducing reliance on costly neutralization assays, its current implementation requires both CDR-H3 and corresponding epitope sequences. These data are often unavailable in high-throughput BCR sequencing and may therefore require experimental determination. To overcome this limitation, integrating Deepstack-NAb with sequence-based or structure-based epitope prediction pipelines could enable accurate neutralization predictions without prior experimental epitope mapping, thereby broadening its applicability. We anticipate that Deepstack-NAb will serve as a valuable tool for the screening and identification of NAbs against DENV-1 to DENV-4, thereby accelerating the development of effective therapeutic antibodies.

## Supplementary Information

Below is the link to the electronic supplementary material.

Supplementary Material 1

## Data Availability

All the data used in this study are available at https://github.com/saeed344/Deepstack-NAb.
