# Supplementary material for: Accurate identification of broadly neutralizing antibodies against dengue virus based on deep stacking strategy with multi-perspective features
Source: Sci Rep. 2025 Dec 10;16:1720. doi: 10.1038/s41598-025-31332-3 (PMC12800099; doi:10.1038/s41598-025-31332-3)
Supplement: Supplementary file 1 — Supplementary Material 1 [file 41598_2025_31332_MOESM1_ESM.docx]

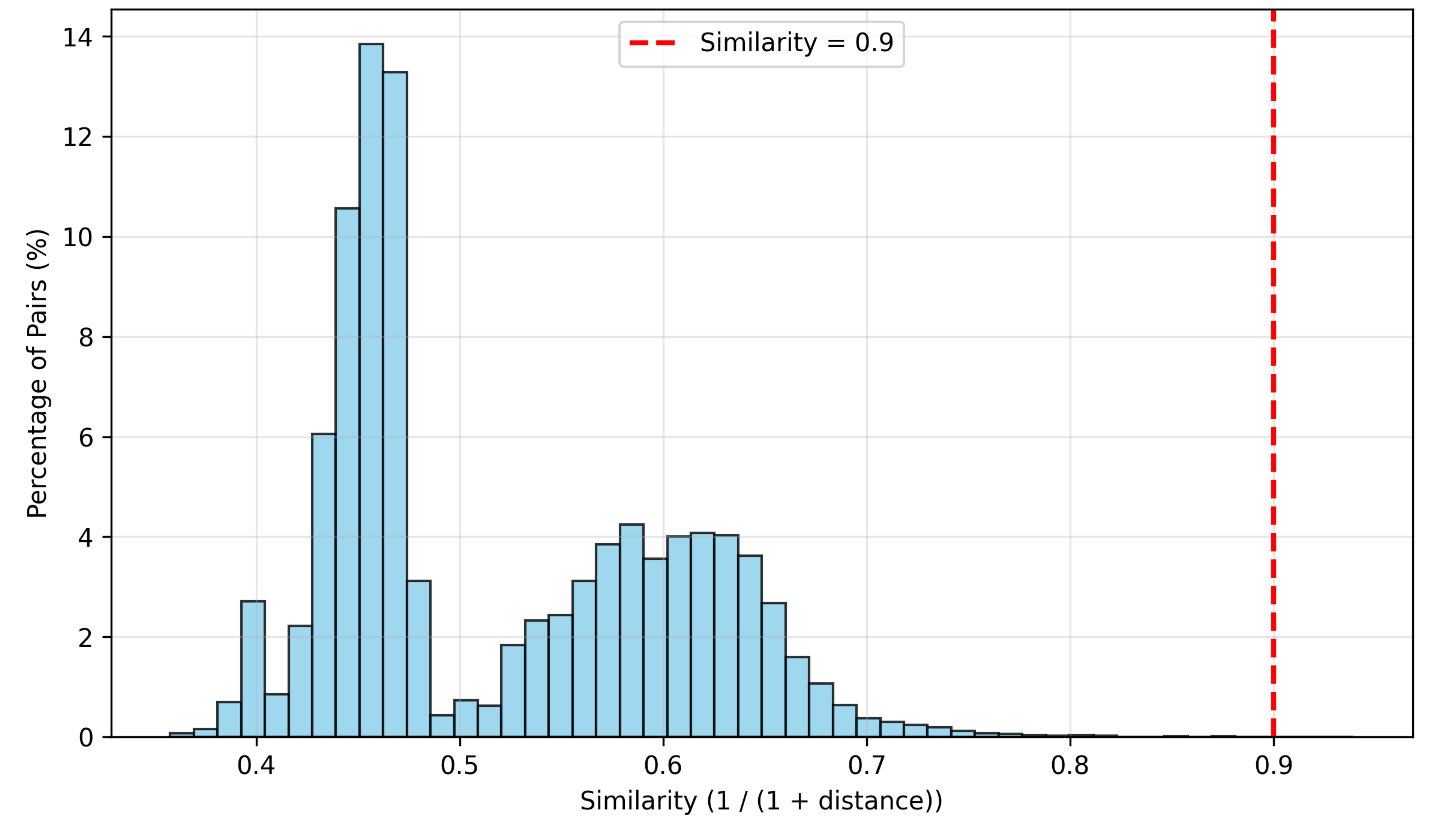

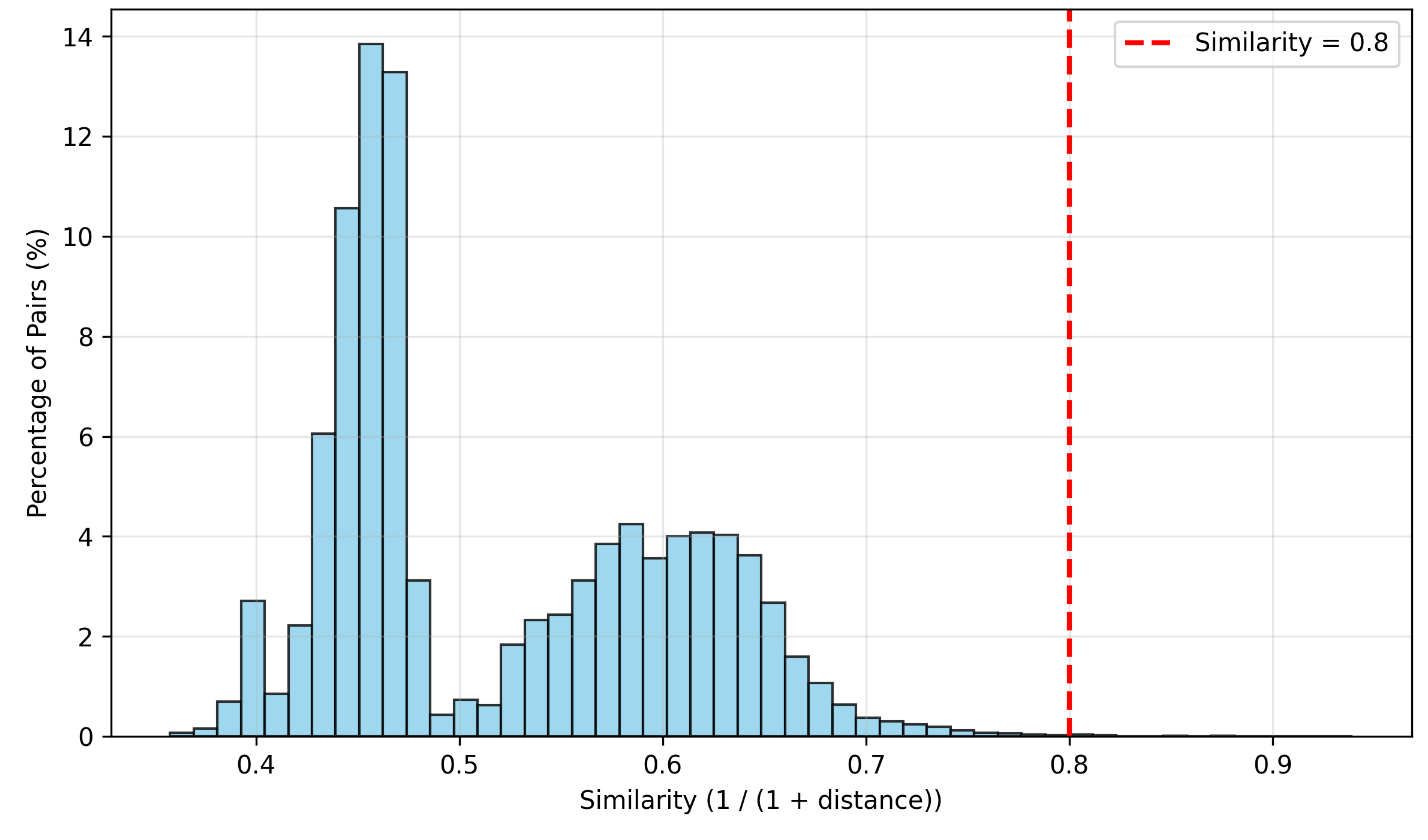


**B**

**A**

**D**

**C**


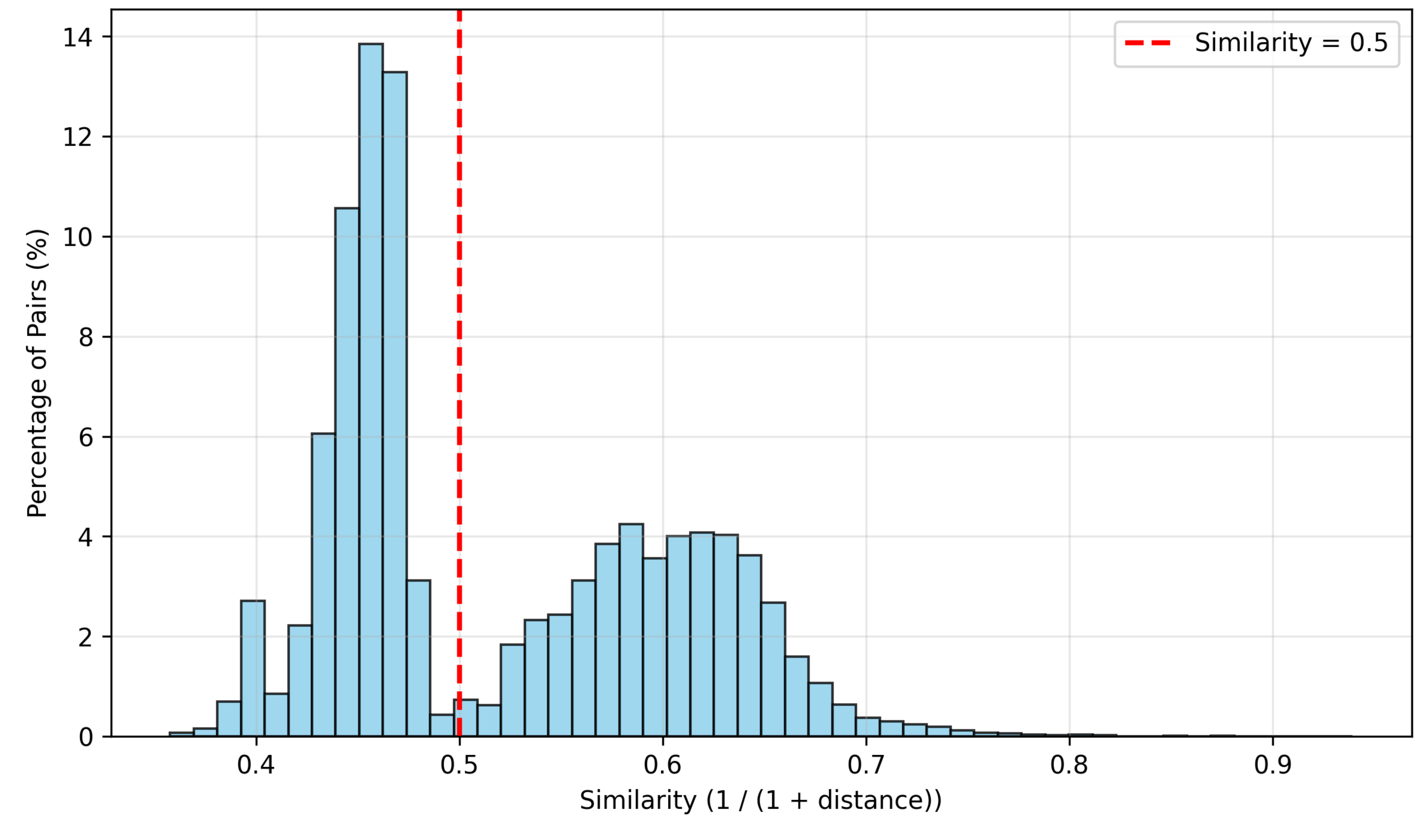


**E**

**Figure S1.**  Histogram of percentage of sequence pairs exceeding multiple similarity cut-offs in the training and independent test datasets. (**A**) < 0.9. (**B**) < 0.8. (**C**) < 0.7. (**D**) < 0.6. (**E**) < 0.5


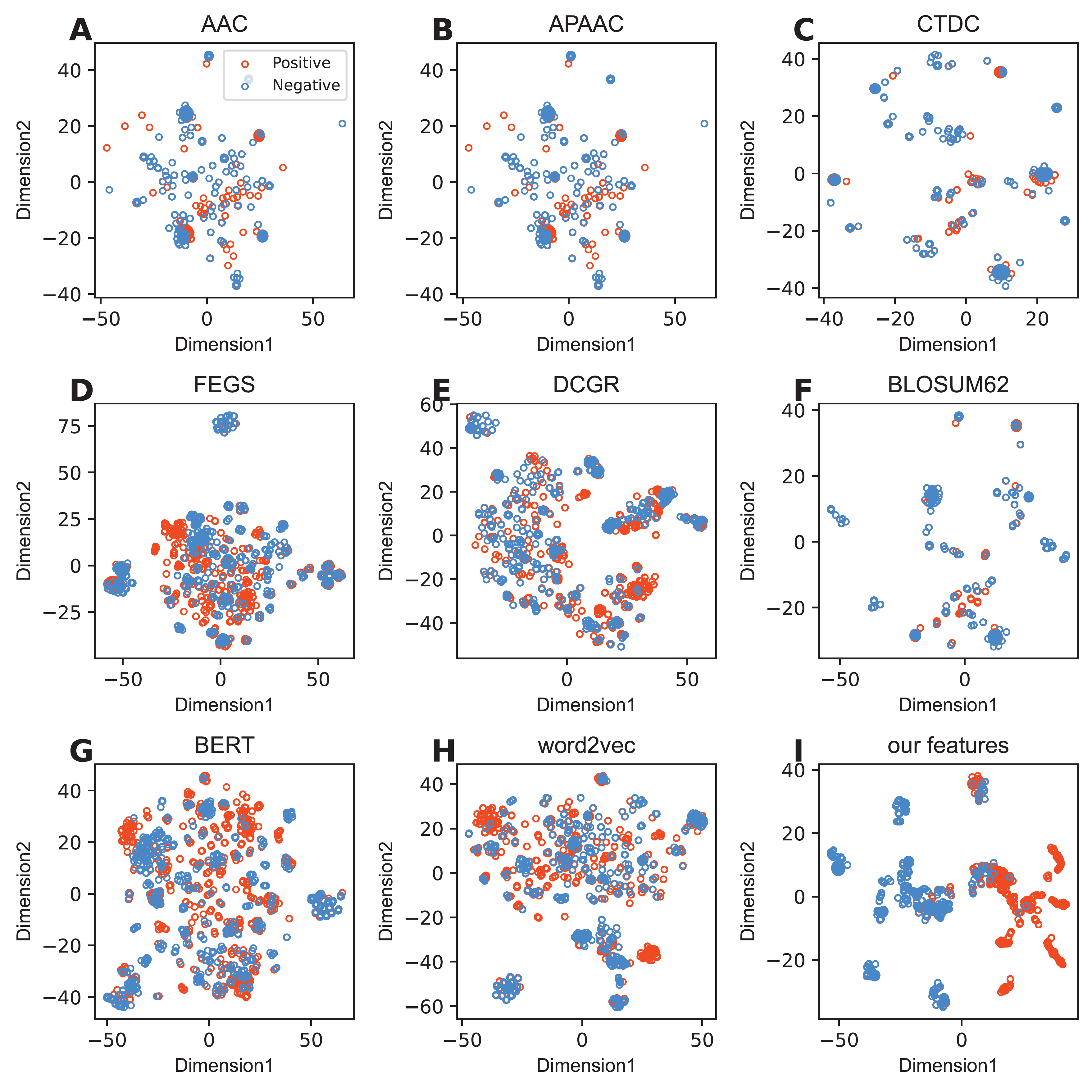


**Figure S2.**  t-SNE visualizations of conventional feature descriptors (i.e., AAC, APAAC, CTDC, FEAGS, DCGR, BLOSUM62, word2vec, and BERT) and our proposed multi-perspective features (i.e., PF) in two-dimensional space.

## Table S1. A summary of number and percentage of sequence pairs exceeding multiple similarity cut-offs in the training and independent test datasets

| **Cut-off** | **Number of pairs** | **Percentage (%)** |
| --- | --- | --- |
| $\geq$ 0.9 | 11 | 0.01 |
| < 0.9 | 196,681 | 99.99 |
| $\geq$ 0.8 | 225 | 0.11 |
| < 0.8 | 196,467 | 99.89 |
| $\geq$ 0.7 | 2,699 | 1.37 |
| < 0.7 | 193,993 | 98.63 |
| $\geq$ 0.6 | 46,948 | 23.87 |
| < 0.6 | 149,744 | 76.13 |
| $\geq$ 0.5 | 90,226 | 45.87 |
| < 0.5 | 106,466 | 54.13 |

The total number of pairs the training and independent test datasets is 196,692

## Table S2. Information of parameter settings for ML and DL methods used in this study.

| **Method** | **Parameter** | **Search space** |
| --- | --- | --- |
| ET | n_estimators | [20, 50, 100, 200, 500] |
| MLP | hidden_layer_sizes | [20, 50, 100, 200, 500] |
| RF | n_estimators | [20, 50, 100, 200, 500] |
| SVM | Cost | [2^-4^–2^4^] in log_2_ steps |
| XGB | n_estimators | [20, 50, 100, 200, 500] |
| DNN | hidden_layers | [1, 2, 3, 4] |
|  | units_per_layer | [16, 32, 64, 128, 256] |
|  | activation | ['relu', 'sigmoid', 'tanh'] |
|  | dropout_rate | [0.2, 0.3, 0.4, 0.5] |
| GRU | units | [16, 32, 64, 128] |
|  | dropout_rate | [0.2, 0.3, 0.4, 0.5] |
|  | activation | ['relu', 'sigmoid', 'tanh'] |
| CNN | Filters (Conv1D) | [16, 32, 64, 128, 256] |
|  | Kernel Size | [3] |
|  | Activation | ['relu', 'sigmoid', 'tanh'] |
|  | Optimizer | ['adam', 'sgd', 'rmsprop', 'adamax'] |
|  | Learning Rate | [0.001, 0.01, 0.1] |
|  | Dropout Rate | [0.0, 0.2, 0.3, 0.5] |

Columns 2 and 3 represents the parameter name used in the Scikit-learn library and the range of parameter used to develop the model, respectively.
